# The Effect of Calcium Ions on hIAPP Channel Activity: Possible Implications in T2DM

**DOI:** 10.3390/membranes13110878

**Published:** 2023-11-09

**Authors:** Daniela Meleleo, Giuseppe Cibelli, Anna Valenzano, Maria Mastrodonato, Rosanna Mallamaci

**Affiliations:** 1 Department of Science of Agriculture, Food, Natural Resources and Engineering, University of Foggia, 71122 Foggia, Italy; 2 Department of Clinical and Experimental Medicine, University of Foggia, 71122 Foggia, Italy; giuseppe.cibelli@unifg.it (G.C.); anna.valenzano@unifg.it (A.V.); 3 Department of Biosciences, Biotechnologies and Environment, University of Bari “Aldo Moro”, 70125 Bari, Italy; maria.mastrodonato@uniba.it (M.M.); rosanna.mallamaci@uniba.it (R.M.)

**Keywords:** calcium, hIAPP, ion channel, spherical oligomers

## Abstract

The calcium ion (Ca^2+^) has been linked to type 2 diabetes mellitus (T2DM), although the role of Ca^2+^ in this disorder is the subject of intense investigation. Serum Ca^2+^ dyshomeostasis is associated with the development of insulin resistance, reduced insulin sensitivity, and impaired glucose tolerance. However, the molecular mechanisms involving Ca^2+^ ions in pancreatic β-cell loss and subsequently in T2DM remain poorly understood. Implicated in the decline in β-cell functions are aggregates of human islet amyloid polypeptide (hIAPP), a small peptide secreted by β-cells that shows a strong tendency to self-aggregate into β-sheet-rich aggregates that evolve toward the formation of amyloid deposits and mature fibrils. The soluble oligomers of hIAPP can permeabilize the cell membrane by interacting with bilayer lipids. Our study aimed to evaluate the effect of Ca^2+^ on the ability of the peptide to incorporate and form ion channels in zwitterionic planar lipid membranes (PLMs) composed of palmitoyl-oleoyl-phosphatidylcholine (POPC) and on the aggregation process of hIAPP molecules in solution. Our results may help to clarify the link between Ca^2+^ ions, hIAPP peptide, and consequently the pathophysiology of T2DM.

## 1. Introduction

Amyloid disorders are a group of protein-misfolding diseases in which the different proteins—amylin or islet amyloid polypeptide (IAPP), beta peptide (Aβ), prion protein, and synuclein—share the characteristic of aggregating into soluble oligomers, which evolve toward insoluble aggregates, ultimately leading to the formation of mature fibrils. Although there is no sequence homology between the different proteins, the aggregation process follows a nucleation-dependent polymerization process [1,2]. Furthermore, recent studies have shown that some peptides are able to polymerize with other proteins, forming heteropolymers. The IAPP_37_–Aβ42 heterocomplex is an example of heteropolymerization and represents the link between Alzheimer’s disorder (AD) and type 2 diabetes mellitus (T2DM) [3], which are classified as amyloid diseases [4].

Human islet amyloid polypeptide (hIAPP) is a hormone synthesized by the pancreatic islet β-cells, stored in the secretory granule, and secreted into the blood together with insulin. hIAPP is a peptide consisting of 37 amino acids in its native form; it has a disulfide bridge involving the amino acids Cys2 and Cys7, a highly hydrophobic amidated C-terminal (amino acid sequence 30–37) and an amino acid sequence (residues 23–29) that appears to be involved in the initiation of the aggregation process [5,6]. hIAPP, like other peptides responsible for amyloid disorders, shows a strong tendency to self-assemble into β-sheet-rich aggregates that evolve toward amyloid deposits. Both have been found in the extracellular space of the pancreatic islets and seem to be associated with reduced mass and volume among pancreatic islet β-cells [7]. Pathophysiological studies have shown the link between amyloid deposits, β-cell loss, and T2DM [8,9,10,11]. In white type 2 diabetes patients, the amyloid deposits in islets exceed 80%, and they appear to be the pathological changes in T2DM. Furthermore, amylin aggregates appear to drive macrophage-elicited inflammation, leading to β-cell dysfunction [12,13] and suppressing α-cell increase [9,14,15], suggesting that they are not only toxic to β-cells but also to other islet cells [8]. The amylin fibrillation process is a dynamic phenomenon that, through the formation of soluble oligomers, which in turn evolve toward insoluble aggregates and protofibrils, leads to the formation of mature fibrils. The results of several studies show that small soluble oligomers are more toxic than mature fibrils. Numerous studies indicate that different types of aggregates (from small soluble aggregates to mature fibrils) may be implicated in the decline in the β-cell functions until their death through multiple mechanisms of action, as reported by Cao and colleagues [16]. Mature fibrils and amyloid deposits may be responsible for β-cell apoptosis and dysfunction, although the mechanism has not been fully elucidated, while soluble oligomers appear to be responsible for the disruption or permeabilization of the cell membrane due to their ability to interact with bilayer lipids. An unregulated influx of ions into the cell can be produced as a result of the direct interaction of hIAPP oligomers with the membrane. This interaction can occur through the formation of ion channels or in a detergent-like mode [17]. Alternatively, hIAPP oligomers may engage with different membrane receptors, including the amylin receptor, CT, and RAMP receptor [18,19,20]. Many factors can influence hIAPP toxicity by modulating its aggregation process. Peptide concentration [21], electrostatic and/or hydrophobic interactions [22,23], pH [22,24], temperature [25,26], and metal ions [27,28] are some of the factors extensively investigated. Numerous studies have shown that amylin can bind to different metal ions, and the amino acids responsible for binding with metal ions are His18 and Lys1. The interaction of zinc ion, Zn^2+^ [29,30,31,32]; copper ion, Cu^2+^ [33,34,35,36]; aluminum ion, Al^3+^ [37,38]; iron ion, Fe^2+^, in the form of heme [39]; cadmium ion, Cd^2+^; mercury ion, Hg^2+^ [40]; gold ion, Au^3+^ [41]; ruthenium ion, Ru [42,43,44,45]; and vanadium ion, V [46,47] with the hIAPP molecule affects its secondary structure and subsequently its capacity to aggregate. Calcium ion (Ca^2+^) is a metal ion involved in important physiological actions such as muscle contraction, excitability, and the regulation of several intra- and extracellular transfer processes, but it can also become toxic for the cell. Several studies have shown a link between Ca^2+^ and T2DM, although the results obtained seem to be contradictory. According to some researchers, the increased serum Ca^2+^ concentration could be a risk factor for developing T2DM, while others see no correlation. Other studies have shown that serum Ca^2+^ dyshomeostasis is associated with the development of insulin resistance, reduced insulin sensitivity, and impaired glucose tolerance, all hallmarks of T2DM [48]. Furthermore, in the 1990s, different groups of researchers observed that in different tissues from diabetic patients [49,50] or from animal models [50], intracellular concentrations of Ca^2+^ were higher than physiological ones, together with a decrease in the activity of Ca^2+^ ATPase [50]; indeed, many symptoms associated with diabetes can be attributed to the misregulation of Ca^2+^ cellular homeostasis [51].

To elucidate the molecular mechanisms involving Ca^2+^ in pancreatic β-cell loss and subsequently in T2DM, several studies have been conducted, although these mechanisms remain poorly understood. The results of some studies indicate that Ca^2+^ favors an abnormal interaction of hIAPP with negatively charged lipid vesicles, and consequent to this interaction, the peptide changes its conformation from α-helix to β-sheet. Ca^2+^ changes the hIAPP-mediated membrane destruction mechanisms by hindering the insertion of the prefibrillar hIAPP aggregates into the hydrophobic core of the membrane and favoring its destruction with a detergent-like mechanism. According to the authors, the effect of Ca^2+^ on membrane permeabilization by hIAPP is due to the effects of the ion on the biophysical characteristics of the bilayer, although they do not exclude the inhibitory effect of the ion on fibril formation as obtained from the thioflavin T (ThT) assay [52]. Wang and collaborators evaluated the effect of some divalent ions, namely Ca^2+^, Zn^2+^, and Cu^2+^, on the aggregation of both the full-length hIAPP and hIAPP18-27 fragment and the ability of their oligomers to damage the membrane of unilamellar vesicles comprising a mixture of POPC:POPG = 4:1. The aggregation of the hIAPP18-37 fragment is little affected by Ca^2+^ ions when the peptide–metal ion ratio is 1:1 in phosphate buffer pH 7.4. Under the same experimental conditions, the ThT fluorescence intensity emitted by the full-length hIAPP oligomers in the presence of Ca^2+^ is slightly lower than that obtained in its absence, indicating that aggregation is little affected by the ion. The ability to damage the membrane was assessed in terms of the release of calcein from the vesicles. This ability is higher for the peptide fragment or the full-length peptide in the absence of Ca^2+^, indicating that the ion reduces the ability of the peptide to damage the membrane, most likely by reducing the surface hydrophobicity of the oligomers [53].

This study aimed to evaluate the effect of Ca^2+^ ions, at hIAPP:Ca^2+^ molar ratios of 1:1, 1:5, and 1:10 on the aggregation process of the peptide in solution, using the ThT fluorescence assay and transmission electron microscopy (TEM) techniques, as well as the effect on the ability of the peptide to incorporate and form ion channels in zwitterionic planar lipid membranes (PLMs) composed of Palmitoyl-oleoyl-phosphatidylcholine (POPC), using the electrophysiological technique of single-channel current measurements.

## 2. Materials and Methods

### 2.1. Chemicals

Calcium chloride (CaCl_2_), potassium chloride (KCl), thioflavin T (ThT), 1,1,1,3,3,3-hexafluoro-2-propanol (HFIP), and n-decane were purchased from Sigma (Munich, FRG); palmitoyl-oleoyl-phosphatidylcholine (POPC) was purchased from Avanti Polar Lipid (Alabaster, AL, USA). Amylin or hIAPP was purchased from Alexotech (Umeå, Sweden).

### 2.2. Preparation of hIAPP and CaCl_2_ Solutions

To obtain a disaggregated peptide, a 1 mg quantity of hIAPP was dissolved in hexafluoroisopropanol (HFIP) and allowed to incubate at room temperature (RT) for 6 h. Subsequently, the solution was divided into equal portions, with each aliquot being subjected to freezing at −80 °C for 24 h followed by overnight lyophilization. The resulting lyophilized samples were then stored at −20 °C until they were ready for use.

For the single-channel measurement experiments, the lyophilized sample was solubilized in sterile bidistilled water via stirring for 5 min, resulting in a concentration of 256 µM. From this solution, 5 µL was extracted and diluted in 45 µL of sterile bidistilled water via stirring for 5 min, resulting in a concentration of 25.6 µM. A stock solution of CaCl_2_ was prepared by dissolving CaCl_2_ powder (1.5 mg) in 5 mL of sterile bidistilled water via stirring for 10 min, resulting in a concentration of 2 mM. The 1 and 0.1 mM CaCl_2_ solutions were prepared via scalar dilution from a stock solution. These solutions were then refrigerated at 4 °C until needed.

For the TEM and ThT experiments, the lyophilized samples were dissolved in phosphate-buffered saline (PBS 50 mM at pH 7.4), achieving concentrations of 33 µM and 10 µM immediately prior to the TEM and ThT measurements, respectively. The stock solution of CaCl_2_ was prepared by dissolving CaCl_2_ powder (0.73 g) in 5 mL of sterile bidistilled water via stirring for 10 min to obtain a concentration of 1 M. The CaCl_2_ solutions at concentrations of 0.1 M, 1, 0.375, 1.875, and 3.750 mM were obtained from the stock solution via scalar dilution. The CaCl_2_ solutions were stored at 4 °C until use.

### 2.3. Single-Channel Measurement

The investigation into hIAPP’s capacity to incorporate and form ion channels was carried out in planar lipid membranes (PLMs) composed of POPC in 1% n-decane, as detailed in prior descriptions [54,55,56].

The method used in the single-channel measurements was consistent with our earlier studies [40]. To elaborate briefly, PLMs were established spanning a 300 µm aperture within a Teflon partition, which separated two Teflon chambers, each with a volume of 4000 µL. These chambers were referred to as the *cis* and *trans* sides. The *cis* side, containing the hIAPP solution, defined the polarity of the voltage. A negative potential value was applied to the *trans* side, opposite the *cis* side. The two chambers held symmetrical 0.1 M KCl solutions, with pH = 7 and temperature 23 ± 1 °C.

Analytical-grade salts were employed in all experimental procedures. Planar lipid membranes (PLMs) were formed using the Müller–Rudin or painted technique, following previously established protocols [57,58,59]. To monitor the membrane current, we utilized the experimental setup outlined by Meleleo [60]. Subsequently, the recorded data on a chart recorder were manually analyzed. Membrane capacitance was determined through the application of a calibration curve, as detailed in the work of Micelli et al. [61]. For single-channel recordings, the data, filtered at 300 Hz in line with previous studies [62], were collected from a minimum of four experiments conducted on separate days.

To evaluate the effect of the preincubation at RT for 24 h on hIAPP channel activity, two series of experiments were performed:

- In the first experimental set, we added 15.6 µL of freshly prepared hIAPP solution (25.6 µM) to the *cis* side;
- In the second experimental set, 15.6 µL of hIAPP solution (25.6 µM) preincubated for 24 h at RT was added to the *cis* side of the membrane.

The final concentration of the peptide used in this study was 0.1 µM.

To investigate the impact of varying Ca^2+^ concentrations on the peptide in solution, the following experimental series was devised:

- In the initial set, 5 μL of CaCl_2_ (0.1 mM) was combined with 20 μL of hIAPP (25.6 µM), resulting in an equal molar ratio of hIAPP to Ca^2+^ (1:1);
- In the second set, 2.3 μL of CaCl_2_ (1 mM) was combined with 17.7 μL of hIAPP (25.6 µM), resulting in a molar ratio of hIAPP to Ca^2+^ of 1:5;
- In the third set, 2.3 μL of CaCl_2_ (2 mM) was combined with 17.7 μL of hIAPP (25.6 µM), resulting in a molar ratio of hIAPP to Ca^2+^ of 1:10.

After preparation, each mixture of metal ions and peptides was stirred for 3 min. Subsequently, in each experimental set, hIAPP was subjected to a 24 h preincubation with metal ions at RT. Following the formation of the lipid bilayer, 19.60 µL of the first mixture or 17.60 µL of the second and third mixtures, also stirred for 3 min, was introduced into the *cis* side of the membrane. This yielded a final hIAPP concentration of 0.1 µM, combined with the following metal ion concentrations:

- In the initial set, the CaCl_2_ concentration was 0.1 µM;
- In the second set, the CaCl_2_ concentration was 0.5 µM;
- In the third set, the CaCl_2_ concentration was 1 µM.

The parameters considered in this study to evaluate the channel activity of hIAPP in the absence and presence of Ca^2+^ were its conductance, lifetime, frequency, and size of the single channel.

To determine the conductance, we first measured, by hand, the current amplitudes of the single channels, which revealed the existence of one main conductance level. We then constructed a histogram of the amplitude distribution and fitted it with a Gaussian distribution function, giving the central value of the single-channel conductance. The results are expressed as central conductance ± standard error (Λ_c_ ± SE) and were evaluated using analysis of variance (ANOVA–Tukey test) and Student’s *t*-test.

To determine the frequency, we first counted all the channel events, i.e., the openings from the baseline current, and then we calculated the frequency. The frequency represents the mean number of openings in a period of 1 min, obtained from a total number of records. The results are expressed as frequency ± standard deviation (F ± SD).

To calculate the size of the hIAPP channel, the following formula was used:

(1) $$\Lambda_{c}=\frac{\left(\sigma \times \pi \times r^{2}\right)}{d}$$

where Λ*_c_* is the central conductance, *σ* is the specific conductibility of the solution filling the channel, *π* has its usual meaning, *r* is the radius of the channel and *d* is its length.

### 2.4. Thioflavin-T Assay

To determine the level of hIAPP amyloid aggregates in the absence and presence of different Ca^2+^ concentrations, the ThT fluorescence assay was used. We set up the hIAPP samples in the absence and presence of the metal ion (called preincubation samples) as follows:

- A 75 µL sample of hIAPP was taken from a stock solution at 10 µM;
- Then, 2 µL of CaCl_2_ at 0.375 mM was combined with 75 μL of hIAPP at 10 µM, yielding a molar ratio of hIAPP to Ca^2+^ of 1:1;
- Next, 2 µL of CaCl_2_ at 1.875 mM was combined with 75 μL of hIAPP at 10 µM, yielding a molar ratio of hIAPP to Ca^2+^ of 1:5;
- Lastly, 2 µL of CaCl_2_ at 3.750 mM was combined with 75 μL of hIAPP at 10 µM, yielding a molar ratio of hIAPP to Ca^2+^ of 1:10.

After preparation, samples were stirred for 1 min and preincubated for 24 or 72 h at RT. Preliminarily, three solutions of CaCl_2_ in PBS at concentrations of 10, 50, and 100 µM were prepared as follows:

- For 10 µM CaCl_2_, 20 µL of 0.1 mM CaCl_2_ (aqueous solution) was added to 980 µL of PBS;
- For 50 µM CaCl_2_, 10 µL of 1 mM CaCl_2_ (aqueous solution) was added to 990 µL of PBS;
- For 100 µM CaCl_2_, 20 µL of 1 mM CaCl_2_ (aqueous solution) was added to 980 µL of PBS.

After the preincubation time, samples were set up for the determination of the fluorescence intensity (called reading samples) as follows:

- In the hIAPP reading sample, 62 µL of hIAPP sample was combined with 246 µL of PBS and 2 µL of ThT (2 mM);
- In the hIAPP + Ca^2+^ (10 µM) reading sample, 62 µL of hIAPP + Ca^2+^ (10 µM) sample was combined with 246 µL of PBS and 2 µL of ThT (2 mM);
- In the hIAPP + Ca^2+^ (50 µM) reading sample, 62 µL of hIAPP + Ca^2+^ (50 µM) sample was combined with 246 µL of PBS and 2 µL of ThT (2 mM);
- In the hIAPP + Ca^2+^ (100 µM) reading sample, 62 µL of hIAPP + Ca^2+^ (100 µM) sample were combined with 246 µL of PBS and 2 µL of ThT (2 mM);
- In the control samples, we combined 308 µL of PBS or 10 µM CaCl_2_, 50 µM CaCl_2_, or 100 µM CaCl_2_ with 2 µL of ThT (2 mM).

In all reading samples, the final concentrations of hIAPP (except in control samples) and ThT were 2 µM and 10 µM, respectively.

A 2 mM ThT solution in PBS was freshly prepared and added to the Eppendorf tubes holding the samples 15 min before instrumental analysis. In a 96-well black plate with a clear bottom, ThT fluorescence intensity was measured using a CLARIOstar microplate reader (BMG Labtech, Ortenberg, Germany) at a temperature of 37 °C and a pH of 7.4. The samples were orbitally shaken for 30 s, with an amplitude of 4 mm and a frequency of 200 rpm. Emission was measured at 480 ± 10 nm and excitation was measured at 440 ± 10 nm with a 10 nm slit; data points were collected at 1 min intervals. Each experiment was carried out in triplicate.

### 2.5. Transmission Electron Microscopy

The morphology of hIAPP aggregates, both in the absence and presence of Ca^2+^, was evaluated using TEM. hIAPP (10 µM) was incubated alone or with 10, 50, and 100 µM CaCl_2_ at RT for 30 min (T0), 24 h (T24), and 72 h (T72).

Initially, CaCl_2_ solutions at concentrations of 10, 50, and 100 µM in PBS were prepared by mixing 1, 5, and 10 µL of CaCl_2_ (1 mM aqueous solution) with 99, 95, and 90 µL of PBS. The samples were prepared as follows:

- In the hIAPP sample, 3 µL of the hIAPP stock solution (33 µM) was combined with 7 µL of PBS, yielding a final concentration of 10 µM;
- In the hIAPP + Ca^2+^ (10 µM) sample, 3 µL of the hIAPP stock solution (33 µM) was combined with 7 µL of CaCl_2_ (10 µM) in PBS, yielding an hIAPP:Ca^2+^ molar ratio of 1:1;
- In the hIAPP + Ca^2+^ (50 µM) samples, 3 µL of the hIAPP stock solution (33 µM) was combined with 7 µL of CaCl_2_ (50 µM) in PBS, yielding an hIAPP:Ca^2+^ molar ratio of 1:5;
- In the hIAPP + Ca^2+^ (100 µM) samples, 3 µL of the hIAPP stock solution (33 µM) was combined with 7 µL of CaCl_2_ (100 µM) in PBS, yielding an hIAPP:Ca^2+^ molar ratio of 1:10.

Subsequently, 10 µL of each sample was blotted onto a carbon-coated Formvar 300 mesh copper grid for 1 min. The excess sample was removed with filter paper, and the grids were washed. The grids were negatively stained with saturated uranyl acetate for 1 min, dried at room temperature, and then observed under a Morgagni 268 electron microscope (FEI, Hillsboro, OR, USA) at a voltage of 70 kV.

### 2.6. Statistical Analysis

Statistical analyses (Gaussian distribution function, ANOVA test, Student’s *t*-test, and fitting procedures) were carried out using GraphPad Prism 3 software (GraphPad Prism^TM^ version 3.0). A value of *p* < 0.05 was considered significant. The minimum and maximum number (N) and total number (N_t_) of individual channel events for each experimental series were reported.

## 3. Results

In this work, the ability of hIAPP to incorporate and form ion channels in PLMs composed of POPC after 24 h preincubation at RT in the absence and presence of varying calcium concentrations was evaluated.

In all experiments, before peptide addition, the PLM was stabilized using voltages of ±80 and ±100 mV consecutively for 10 min each. During the membrane’s stabilization phase, the conductance and capacitance remained at constant values of 26 pS and 0.30 µF/cm^2^, respectively, and no channel activity appeared. After the membrane stabilization step, the addition of the peptide solution or the peptide–calcium mixture was carried out at the voltage of 100 mV (addition voltage). After different lag times, which depended on the experimental conditions in the different sets, the channel activity of hIAPP was observed at an activation voltage of 100 mV as abrupt, discrete, nonrandom current jumps from the baseline current, consistent with the finding of our previous study [40]. The channel activity alternated with periods of quiescence and periods of paroxystic activity whose duration depended on the experimental conditions, as will be discussed in what follows. In the initial phase of hIAPP incorporation into the PLM and during the period of paroxystic activity, current transitions directed upward were more frequent than those directed downward. By applying the same experimental protocol used in our previous study [40], the channel activity was monitored in the range of applied voltages from ±20 to ±100 mV. Briefly, after the appearance of the first channel, the applied voltage was lowered as far as ±20 mV, and the channel amplitude was recorded on the chart recorder. Each voltage was applied for 90 min starting at 100 mV, and then 90 min at −100 mV, and so on.

### 3.1. Effect of 24 h Preincubation on hIAPP Channel Activity

Two sets of experiments were set up to evaluate the effect of preincubation on hIAPP channel activity. In the first set of experiments, a known volume of freshly prepared hIAPP stock solution (25.6 µM) was added into the medium of the *cis* chamber, while in the second experimental set, the same volume of a stock solution of hIAPP preincubated for 24 h at RT was added. In both experimental sets, the final concentration of the peptide was 0.1 µM. After the addition of the stock solutions, the *cis* chamber medium was stirred for 1 min. The channel activity appeared in both experimental sets at the applied voltage of 100 mV after different lag times, which depended on the preincubation treatment.

Preliminarily, we tested membrane stability by performing control experiments with the lipid mixture only to exclude nonspecific effects. No changes in the parameters of capacitance (0.30 µF/cm^2^) and conductance (26 pS) were observed for a time of 5 h at the applied voltages of ±100 mV.

In the first experimental set, the channel activity appeared after 3 h as current fluctuations, compatible with channel-type openings and closings, showing different levels of conductance (channel activity). As described above, once the first channel appeared, the voltage was reduced to ±20 mV, and channel amplitude was measured. In this experimental set, the channel activity occurred in the range of applied voltages from ±20 mV to ±100 mV. At all applied voltages, there were periods of quiescence alternating with periods of channel activity.

**Figure 1:**
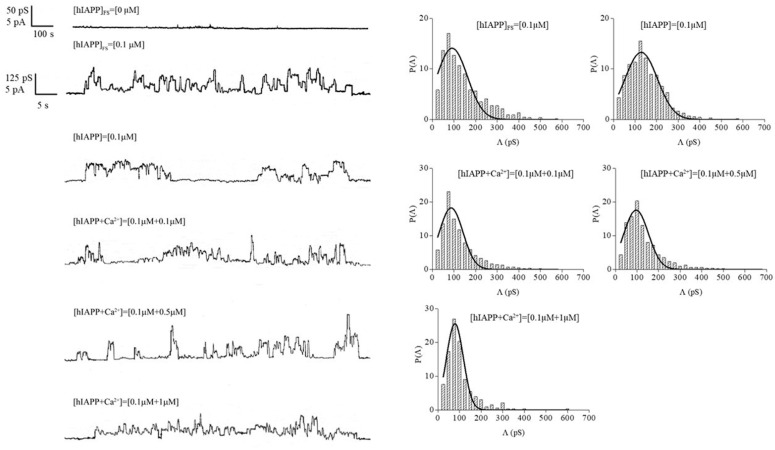
hIAPP channel activity in POPC PLMs with associated histograms of the independent channel events’ conductance fluctuations. Chart recordings of the channel activity of hIAPP in the absence of peptide (control experiment), without preincubation ([hIAPP]_FS_), and with preincubation for 24 h in the absence ([hIAPP]), and in the presence of different concentrations of CaCl_2_ ([hIAPP + Ca^2+^]). Channel activity was recorded at an applied voltage of 40 mV. The voltage applied in the control experiment was 100 mV. The histograms of the probability, P (Λ), for the frequency of a given conductivity unit were fitted using a Gaussian function, which is shown as a solid curve. Experimental conditions: KCl 0.1 M (pH 7) and T = 23 ± 1 °C.

In the second experimental set, the channel activity appeared after membrane rupture, preceded by a decrease in capacitance from the initial value of 0.30 µF/cm^2^ (immediately after the addition of the preincubated stock solution) to the value of 0.20 µF/cm^2^ (about 1 h after adding the hIAPP solution). Immediately after breakage, the PLM was promptly reconstituted with the operator at a voltage of 100 mV, and channel activity appeared after approximately 45 min, manifesting in some experiments as channel activity and in others as paroxystic activity that gradually disappeared and after short periods of quiescence appeared as channel activity. In this set of experiments, channel activity alternated with long periods of quiescence, and this behavior was observed at all applied voltages ranging from ±40 mV to ±100 mV. At applied voltages of ±20 mV, no channel activity was registered for long periods even after the membrane was broken and withdrawn using the operator. Figure 1 shows examples of chart recordings of a control experiment and the hIAPP channel activity in the two different experimental conditions.

**Figure 2:**
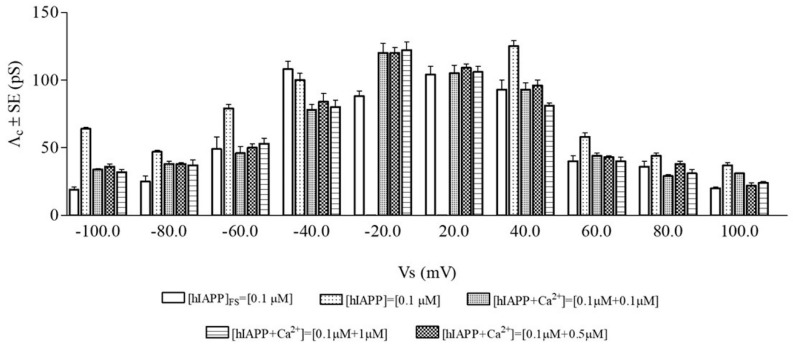
Central conductance (Λ_c_) of hIAPP channel in POPC PLMs. Λ_c_ values (Λ_c_ ± SE) of the hIAPP channel obtained after the addition -of the freshly prepared stock solution ([hIAPP]_FS_), -of the solution preincubated for 24 h without ([hIAPP]), and with different concentrations of Ca^2+^ ([hIAPP + Ca^2+^]). The minimum and maximum number of channels (N) considered out of a total number of channels (N_t_) was [hIAPP]_FS_ = 0.1 µM, 311 < N < 1330, Nt = 4669; [hIAPP] = 0.1 µM, 127 < N < 1260, Nt = 4751; [hIAPP + Ca^2+^] = 0.1 µM + 0.1 µM, 247 < N < 1950, Nt = 7313; [hIAPP + Ca^2+^] = 0.1 µM + 0.5 µM, 259 < N< 1643, Nt = 9126; and [hIAPP + Ca^2+^] = 0.1 µM + 1 µM, 285 < N < 1374, Nt = 5247.

Figure 2 shows the central conductance (Λ_c_ ± SE) at the different applied voltages. It is important to note that, at all applied voltages where comparison is possible, Λ_c_ values obtained after peptide preincubation are significantly higher than those obtained without preincubation except at the applied voltage of −40 mV.

In Formula (1), replacing the value of Λ_c_ at a voltage of 40 mV, obtained in the two different experimental conditions, and assuming a cylindrical channel with length (*d*) 5 nm (corresponding to the membrane thickness) filled with a solution having the same specific conductivity as the external medium, we calculated the channel diameters of hIAPP, which were 6.8 and 7.9 Å without and with preincubation, respectively.

**Figure 3:**
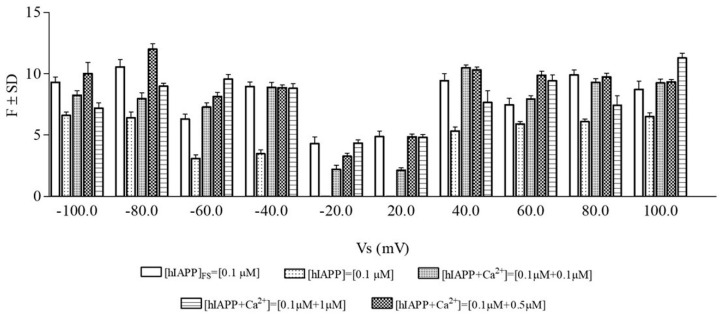
Frequency (F) of hIAPP channel in POPC PLMs. F values (F ± SD) of the hIAPP channel obtained after the addition -of the freshly prepared stock solution ([hIAPP]_FS_), -of the solution preincubated for 24 h without ([hIAPP]), and with different concentrations of Ca^2+^ ([hIAPP + Ca^2+^]). The minimum and maximum number of channels considered (N) out of the total number of channels considered (N_t_) is reported in the legend in Figure 2.

The channel frequency of hIAPP after 24 h preincubation was significantly lower than that obtained using the freshly prepared peptide stock solution at all applied voltages (Figure 3), indicating that the channel turnover was affected by the preincubation process.

To evaluate the stability of the hIAPP channel in the membrane, we calculated its duration. The duration of the channel ranged from 1.25 to 3.25 s when the peptide was not preincubated, whereas it ranged from 3.5 to 10.25 s when it was preincubated for 24 h, indicating greater channel stability in the membrane.

The results obtained from these two experimental sets highlight that the conductance and duration values of the preincubated hIAPP channel were higher, while the frequency was lower than those obtained without preincubation.

### 3.2. Effect of Different Ca^2+^ Concentrations on hIAPP Channel Activity

To evaluate the impact of calcium ions on the hIAPP peptide’s ability to incorporate and form channels in POPC PLMs, we conducted three sets of experiments. In each set, the peptide was preincubated with varying concentrations of CaCl_2_ for 24 h at RT.

Preliminarily, we tested the stability of the membrane in the presence of different concentrations of CaCl_2_ (control experiments) to exclude the destabilization effects of the Ca^2+^ ions. Control experiments were conducted by adding CaCl_2_ (final concentrations 0.1, 0.5, and 1 µM) into the *cis* chamber medium. The biophysical parameters (conductance and capacitance) of the PLM were monitored at applied voltages of ±80 and ±100 mV for a time of 10–12 h during which the conductance (25 pS) and capacitance (0.31 µF/cm^2^) values remained constant, and no channel activity was recorded.

In preincubation experiments, where the hIAPP:Ca^2+^ molar ratio was 1:1, the channel activity occurred at 100 mV following a lag time of 90 min. The channel activity was monitored in applied voltages ranging from ±20 to ±100 mV. It should be noted that at applied voltages of ±20, ±40, and ±60 mV, the channel activity alternated with periods of quiescence, while at ±80 and ±100 mV, short periods of paroxystic activity alternated with those of channel activity and quiescence. Paroxystic activity, regardless of the applied voltage, progressively disappeared, and channel activity appeared.

In preincubation experiments in which the Ca^2+^ concentration was five times that of the peptide (hIAPP:Ca^2+^ = 1:5), the lag time was lowered to 60 min. The channel activity occurred at a voltage of 100 mV, and after the occurrence of the first channel, the applied voltage could be lowered down to ±20 mV. The characteristics of the channel activity were like those observed in the previous experimental set. with quiescent periods alternating with those with channel activity and paroxystic activity.

In the third experimental set, we monitored the channel activity of hIAPP that had been preincubated with the Ca^2+^ ion at the concentration of 1 µM (hIAPP:Ca^2+^ molar ratio = 1:10). The channel activity appeared at 100 mV following a lag time of about 60 min. Also, in the experiments of this set, the channel activity was recorded in applied voltages ranging from ±20 to ±100 mV, manifesting itself with the alternation of periods of channel activity and periods of paroxystic activity, which led to membrane rupture in some experiments.

Examples of chart recordings of the channel activity in the three different experimental conditions are shown in Figure 1.

As carried out for the experiments in the presence of the peptide alone, we fitted the current experimental data with a Gaussian distribution function yielding the Λ_c_ value, and the channel events counted as successful were used to calculate the frequency. Figure 2 and Figure 3 show the biophysical (Λ_c_ ± SE) and statistical (F ± SD) parameters of the hIAPP ion channel in the presence of the three Ca^2+^ concentrations. It is interesting to note that for each Ca^2+^ concentration, the Λ_c_ values are inversely correlated with the applied voltage, decreasing with increasing applied voltage, as found for the Λ_c_ values of the peptide alone (both without and with preincubation). For each applied voltage, the Λ_c_ values obtained in the presence of the three Ca^2+^ concentrations are not significantly different, and for each Ca^2+^ concentration, the frequency values are symmetrical for the negative and positive applied voltages.

Similar to the two previous experimental sets (without and with preincubation), we calculated the size of the channel and its duration in the presence of the three Ca^2+^ concentrations.

By substituting the values of Λ_c_ at 40 mV in the presence of the three concentrations of Ca^2+^ in Formula (1), and assuming *σ*, *π*, and *d* to be unchanged, the diameter of the channel was 6.8/6.9/6.3 Å when the preincubation concentrations of the ion were 0.1/0.5/1 µM, respectively.

The channel duration was in the range of 1.25–5.25 s/1.25–3.25 s/4.25–10.25 s when the peptide was preincubated with 0.1/0.5/1 µM Ca^2+^, respectively. These results indicate greater hIAPP channel stability when preincubating with Ca^2+^ at 1 µM than with preincubation using the other two concentrations of the metal ion.

The results using the electrophysiological technique showed that the Λ_c_ values in the presence of 0.1, 0.5, and 1 µM of Ca^2+^ were significantly lower than those of the peptide preincubated 24 h at all applied voltages; the frequency values, at any ion concentration, were far higher than when preincubating only the peptide; the channel diameter values calculated in the presence of Ca^2+^ were lower than those calculated with the preincubated peptide alone; and the duration of the hIAPP channel depended on the metal ion concentration being lower/similar to that obtained with the preincubated peptide alone when the Ca^2+^ concentrations were 0.1 and 0.5/1 µM, respectively. On the other hand, the biophysical and statistical parameters of the hIAPP channel obtained in the presence of Ca^2+^, regardless of the ion concentration used, were similar to those obtained by using the freshly prepared hIAPP solution.

### 3.3. Effect of Different Ca^2+^ Concentrations on hIAPP Amyloid Aggregate Levels

hIAPP channel frequency values were far higher when preincubating the peptide with the metal ion (regardless of the Ca^2+^ concentration used) than those obtained by preincubating the peptide alone, indicating that preincubation with metal ions favored the formation of aggregates prone to incorporation into the membrane, leading to the formation of ion channels. To verify this result, we conducted fluorescence experiments using the ThT fluorophore. ThT, interacting with the β-sheets of amyloid aggregates, is useful for monitoring the levels of amyloid aggregates and following the state of protein aggregation. We conducted two sets of experiments to measure levels of amyloid aggregates formed by the hIAPP peptide in the absence and presence of varying Ca^2+^ concentrations after 24 and 72 h of incubation.

**Figure 4:**
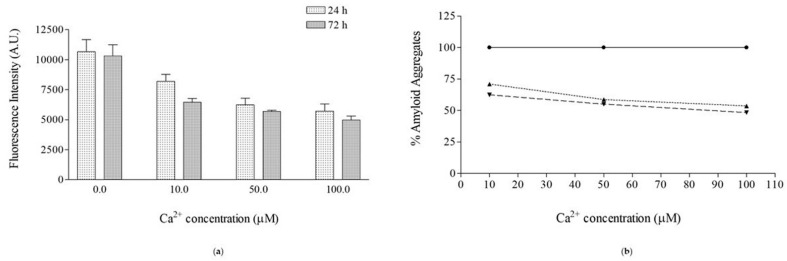
hIAPP amyloid aggregates levels in the absence and presence of different concentrations of CaCl_2_. (**a**) Fluorescence intensity was measured after 24 and 72 h of incubation in PBS pH = 7.4 at 37 °C. (**b**) Percentage values of the fluorescence intensity of hIAPP (10µM) in the absence (●) and in the presence of the three Ca^2+^ concentrations after 24 h (▲) and 72 h (▼) incubation.

Figure 4a shows that the fluorescence intensity decreases as a function of increasing Ca^2+^ concentrations, independently of the incubation time. Notably, the fluorescence intensities obtained by incubating the peptide alone for 24 and 72 h were not significantly different.

Figure 4b reports the percentage fluorescence intensity of the hIAPP peptide in the absence and presence of the three Ca^2+^ concentrations.

The percentage values of the fluorescence intensity, after 24/72 h of incubation, were suppressed by 23/37, 40/45, and 46/51% in the presence of Ca^2+^ at concentrations of 10, 50, and 100 µM, respectively, indicating that the fluorescence intensity decreased in a concentration-dependent manner.

The results obtained with fluorescence spectroscopy revealed that the hIAPP amyloid aggregate levels decreased when the peptide was incubated with Ca^2+^, indicating that the metal ion inhibited the formation of amyloid aggregates. These results seem to be in line with those obtained with the electrophysiological technique.

### 3.4. Effect of Different Ca^2+^ Concentrations on hIAPP Amyloid Aggregate Morphology

**Figure 5:**
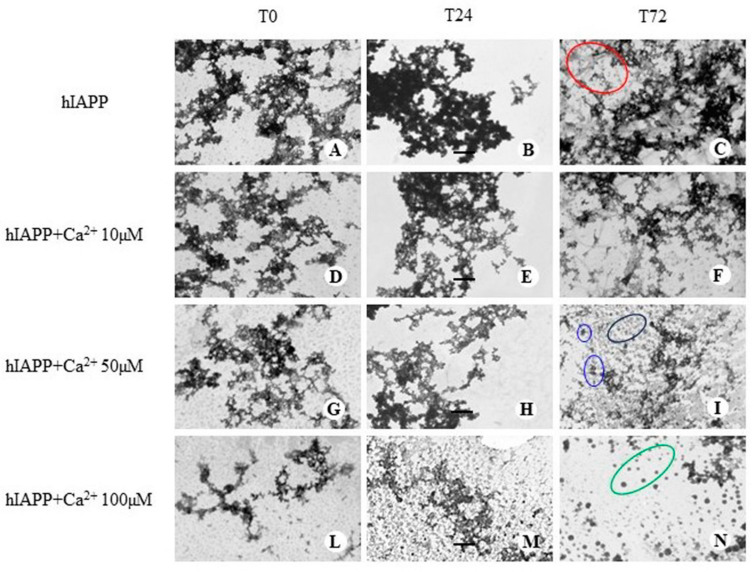
hIAPP aggregate morphology. TEM images of aggregates formed by 10 µM hIAPP alone (**A**–**C**) as well as hIAPP preincubated with the three Ca^2+^ concentrations (**D**,**G**,**L**), (**E**,**H**,**M**) and (**F**,**I**,**N**) for 0 h (T0), 24 h (T24), and 72 h (T72), respectively. A scale bar of 250 nm is provided for reference. These images showcase a variety of structures, including fibrils, short scattered aggregates, low electron-dense spherical structures, and electron-dense spherical structures, which are circled in red, blue, black, and green, respectively.

We performed experiments with the TEM technique to visualize the morphology of aggregates and fibrils formed by hIAPP in the absence and presence of varying Ca^2+^ concentrations after 0, 24, and 72 h of incubation. The TEM images obtained in the presence of the peptide alone (10 µM) show the presence of T0 aggregates, which appear to be more electron-dense after 24 h of incubation; at T72, image C shows the presence of fibrils and aggregates, indicating that the hIAPP aggregation process is a dynamic phenomenon where multiple species can coexist (Figure 5A–C).

The amyloid aggregates obtained in the presence of Ca^2+^ in equimolar quantities with the peptide were not significantly different from those in the absence of the ion at T0 (Figure 5D), while they appeared less electron-dense (Figure 5E,F) than those of the peptide alone after 24 and 72 h of incubation. Furthermore, image F of Figure 5 shows the coexistence of fibrils and aggregates, which are less extensive than those shown in image C. TEM images of the hIAPP + Ca^2+^ 50 µM sample reveal that amyloid aggregates exhibit reduced extent and electron density over the course of 72 h. Image I in Figure 5 (T72) shows the presence of short and scattered aggregates together with low electron-dense spherical structures and the absence of fibrils. The effect of the high concentration of Ca^2+^ (hIAPP:Ca^2+^ molar ratio = 1:10) on the shape of the aggregates is shown in images L, M, and N. It is interesting to note that the amyloid aggregates appear not very extensive and have lower electron density than those formed under the other experimental conditions. Moreover, by T0, small and scattered aggregates can be seen, which decrease in the sample by T24, almost completely disappearing in the sample at T72 where definite and electron-dense spherical structures are present.

The results obtained with electron microscopy indicate that Ca^2+^ incubated with the hIAPP peptide in pH 7.4 phosphate buffer inhibits the formation of fibrils and large aggregates in favor of smaller spherical aggregates. The impact of the ion on the aggregation process seems to be concentration-dependent. Increasing its molar ratio relative to the peptide resulted in the formation of smaller spherical aggregates. These results appear to be in line with those obtained with ThT fluorescence.

## 4. Discussion

Amyloidogenic peptides are a class of small peptides that, while not exhibiting sequence homology, have the common feature of forming insoluble amyloid aggregates and fibrils, which are hallmarks of related disorders. Some examples are the insoluble aggregates and fibrils of the Aβ peptide in AD, α-synuclein in Parkinson’s disorder, prion protein (PrPSc) in Creutzfeldt–Jakob disorder (CJD), and hIAPP peptide in T2DM.

The formation of insoluble aggregates and fibrils in the absence of catalysts follows a nucleation-dependent polymerization reaction. The lag phase is characterized by the presence of monomers, which, in the subsequent phase called homogeneous nucleation, unfavorably change their conformation from random coils to β-sheets and assemble to form “nuclei”, i.e., oligomers of different sizes. In the next phase, called heterogeneous nucleation or elongation, nuclei of different sizes react with the monomers, leading to the formation of high-molecular-weight insoluble oligomers and protofibrils (growth phase), which evolve toward the formation of mature fibrils (plateau phase) [1,2,5]. In the steady state, the quantity of fibrils remains constant and in equilibrium with the previous species. Numerous studies show that the cytotoxicity of amyloidogenic peptides may be attributed to oligomeric intermediates rather than monomers or mature fibrils. The study conducted by Demuro and colleagues on SH-SY5Y cells shows that soluble oligomers of different peptides modify the intracellular Ca^2+^ concentration by altering the permeability of the cell membrane [63]. Some studies have shown that hIAPP amyloid deposits can be present in nondiabetic subjects, while hIAPP soluble oligomers were found to be β-cell toxic, as the study by Camargo et al. shows [64]. Several studies have shown the ability of soluble oligomers to interact with the lipid bilayer damaging the membrane by means of different mechanisms. This may occur through incorporation into the bilayer and the formation of ion channels, as indicated by several authors [65,66,67], and the toxicity of the ion channels can be improved or prevented with Zn^2+^, which reversibly blocks the hIAPP channel at micromolar concentrations, as revealed in the study by Hirakura and collaborators [68]. Other mechanisms include alteration in the fluidity of the membrane or the fragmentation of the membrane like a detergent [69].

The aggregation process of amyloidogenic peptides can be influenced by numerous intrinsic and extrinsic factors, including metal ions. The pro- or anti-aggregating effect of metal ions seems to depend on their molar ratio with the peptides and their chemical characteristics. Zn^2+^, Al^3+^, Fe^2+^, and Cd^2+^ ions were found to promote the formation of insoluble oligomers and protofibrils, while Cu^2+^ and Hg^2+^ ions, Ru and V complexes, and some of the Au^3+^ complexes seemed to inhibit the fibrillation process. Studies of the effect of Ca^2+^ on the hIAPP aggregation process are lacking. The study conducted by Sciacca and collaborators indicates that Ca^2+^ promotes the elongation of hIAPP fibrils in the presence of LUVs composed of POPC:POPS = 7:3 and increases membrane fragmentation in a detergent-like manner while inhibiting the interaction of prefibrillar species with the bilayer of LUVs [52].

In this work, we evaluated the effect of Ca^2+^, at different molar ratios with the peptide, on hIAPP molecules in solution by carrying out experiments in which the peptide was incubated with the Ca^2+^ ions or alone (control experiments). By means of the techniques used in this study, the ability of the peptide to interact with the POPC PLMs, the levels of amyloid aggregates, and their morphology in the absence and presence of the ions were monitored.

The results obtained with the electrophysiological technique show that the peptide preincubated in the absence or presence of different concentrations of Ca^2+^ for 24 h is able to incorporate into POPC PLMs and form ion channels after lag times, which are not significantly different in the experimental conditions used in this study (hIAPP preincubated without or with Ca^2+^). The channel conductance in the absence and presence of metal ions appears to be dependent on the applied voltage, as found in our previous study [40]. The Λ_c_ values of the channel, obtained by preincubating the peptide alone, were higher than those obtained in the presence of Ca^2+^. Conductance is the biophysical parameter correlated with the ionic current passing through the channel, which in turn is correlated with the channel size. The diameter value (7.9 Å) obtained by preincubating hIAPP in the absence of the three concentrations of Ca^2+^ indicates that the conductive unit is larger. Our results seem to agree with those obtained by other authors who have found that the structure of the hIAPP channels, determined in DOPC membranes using molecular modeling and molecular dynamics simulations, consists of three to five oligomeric units assembled in the membrane [17,70,71]. The ability of the hIAPP peptide to incorporate into PLMs and form ion channels has been studied in detail by Mirzabekov and colleagues. This study shows that hIAPP forms voltage-dependent and poorly selective channels in PLMs of different lipid compositions. Channel activity appears to be dependent on lipid composition, ionic strength, and membrane potential [65]. The results obtained by these authors, together with the findings of our study, suggest that channel formation may be one of the mechanisms underlying hIAPP cytotoxicity.

The frequency of the hIAPP channel was significantly lower when the peptide was preincubated alone than when it was preincubated with Ca^2+^, indicating lower turnover of the conductive unit in the PLM. This result indicates that fewer oligomeric structures are prone to incorporate into PLMs and assemble into conductive units. By contrast, the preincubation of the peptide with Ca^2+^ seems to increase the number of oligomeric structures prone to interact with the lipid bilayer. However, it is important to note that the channel frequency values obtained in the presence of the three Ca^2+^ concentrations are not significantly different at all applied voltages, indicating that the hIAPP channel turnover is independent of the metal ion concentration. Our results, albeit indirectly, seem to indicate a nonspecific effect of Ca^2+^ due to its inability to bind to specific amino acids of the primary structure of the peptide, as demonstrated by the results obtained with the CD technique [53,72], as well as its ability to influence the surface hydrophobicity of hIAPP molecules, as shown by the results obtained with 8-anilinonaphthalene-1-sulfonic acid (ANS) binding fluorescence [53]. Several studies indicate that some ions’ pro- or anti-aggregating effect depends on their concentration as they interact with specific amino acids and the stoichiometric peptide–ion ratio can modulate the secondary structure of the peptide. Ca^2+^ ions do not influence the secondary structure of hIAPP [53,72] differently from what was found for the Cu^2+^ ion, which stabilizes the secondary structure of the peptide in a random coil conformation, making the peptide more flexible and less prone to aggregate [36]. On the other hand, the increases in ANS fluorescence tend to decrease as the peptide–metal ion ratio increases from 1/0.33 to 1/1 [53], indicating that Ca^2+^ decreases the hydrophobicity of hIAPP oligomers.

To evaluate the effect of preincubation and Ca^2+^ ions on the channel activity of hIAPP, we performed single-channel experiments by adding a freshly prepared peptide stock solution to the *cis* chamber medium. The channel activity occurred after a lag time of 3 h, longer than the lag times observed in the experiments of the preincubated peptide without and with Ca^2+^, indicating that the molecules of the peptide solubilized immediately before the addition to the *cis* chamber medium must organize into oligomers before incorporating into the PLM and forming ion channels. The conductance and frequency values at the different applied voltages and the channel diameter appear to be more similar to those obtained from preincubation experiments in the presence of Ca^2+^ than to those in the absence of the ion. These results indicate that the lack of preincubation or preincubation with Ca^2+^ favors the incorporation of the peptide into the PLM and the formation of smaller-sized conductive units with a higher frequency than those obtained by preincubating the peptide alone. This result, albeit indirectly, indicates that Ca^2+^, in an aqueous solution with a peptide, helps to stabilize the smallest oligomers prone to interact with the PLM, disfavoring the elongation process.

The results obtained with the electrophysiological technique appear to be in line with those obtained with the ThT assay. The formation of amyloid aggregates was inhibited by Ca^2+^ ions, as found by Sciacca and colleagues, who showed that Ca^2+^ did not significantly reduce the rate of fibril formation [72]. A similar result was obtained by Wang and collaborators, who showed that the weak inhibitory effect of Ca^2+^ ions on the aggregation process tended to increase, even if not significantly, by increasing the peptide–metal ion ratio from 1/0.33 to 1/1 [53]. However, possible effects of the Ca^2+^ ion on the interaction of ThT with the hIAPP peptide cannot be excluded.

Our results show that the antiplatelet effect of Ca^2+^ depends on the incubation time more than on the peptide–Ca^2+^ molar ratio. Indeed, after 72 h of incubation, the values of the fluorescence intensity percentage obtained in the presence of the three concentrations of Ca^2+^ were not significantly different from each other. This result seems to confirm the nonspecific effect of Ca^2+^, as the results of CD [53,72] and single-channel current measurements indicate. We studied the morphology of hIAPP aggregates in the absence and presence of different Ca^2+^ concentrations. The peptide incubated alone forms amyloid aggregates which over time evolve toward the formation of fibrils coexisting with electron-dense amyloid aggregates (Figure 5A–C). Similar results were obtained by other authors [73,74,75]. The TEM images obtained in the presence of Ca^2+^ show that the morphology of the aggregates changes drastically, passing from a fibrillar morphology and amyloid aggregates at 10 µM of Ca^2+^ to an approximately spherical morphology and with small, low electron-dense aggregates at 100 µM of Ca^2+^ after 72 h of incubation (Figure 5D–N). The spherical morphology is indicative of the formation of low-molecular-weight oligomeric species, as found by Wang et al. in the study of the morphology and size distribution of oligomers formed from a truncated form of the hIAPP peptide (hIAPP18-27) in the absence and presence of Ca^2+^, Zn^2+^, and Cu^2+^ [53]. Abedini and colleagues reported the morphology of the soluble intermediates and prefibrillar species of full-length hIAPP, which are similar to those we observed in the peptide samples incubated with Ca^2+^ at concentrations of 50 and 100 µM [75]. Considering our results together with those of the other authors, it is possible to hypothesize that Ca^2+^ stabilizes hIAPP aggregates in the form of low-molecular-weight oligomers.

The results obtained in this study show a link between Ca^2+^ and T2DM; they support the notion that Ca^2+^ could be a risk factor in T2DM as the metal ion promotes the stabilization of small aggregates, which are more cytotoxic than mature fibrils.

## 5. Conclusions

The results obtained in this study using different investigation techniques revealed that the levels of amyloid aggregates decreased when the peptide was incubated with Ca^2+^; the morphology of the aggregates observed at the concentrations of Ca^2+^ 50 and 100 µM was significantly different from that obtained by incubating the peptide alone or at the metal ion concentration of 10 µM; the channel frequency values obtained in the presence of Ca^2+^ were significantly higher than those obtained by preincubating the peptide alone; and the biophysical and statistical parameters obtained by preincubating the peptide with Ca^2+^ were similar to those found using the freshly prepared peptide solution.

The results obtained with the ThT assay and TEM indicate that Ca^2+^ influences the hIAPP aggregation process in solution, and those obtained with the electrophysiological technique indicate that the effect of the metal ion is nonspecific since no correlation was found between the biophysical parameters and the Ca^2+^ concentration in the concentration ranges used in this study.

Our results, albeit indirectly, seem to support and strengthen the hypothesis that Ca^2+^ affects the surface hydrophobicity of hIAPP by stabilizing it in the form of small aggregates rather than interacting with specific amino acids of its primary structure, as reported in the literature.

The results obtained in this study may help to clarify the link between Ca^2+^ ions, hIAPP peptide, and consequently the pathophysiology of T2DM.

## Data Availability

Data are contained within the article.
